# Supplementary material for: Critical mingling and universal correlations in model binary active liquids
Source: Nat Commun. 2017 Jun 28;8:15969. doi: 10.1038/ncomms15969 (PMC5493750; doi:10.1038/ncomms15969)
Supplement: Supplementary Information [file ncomms15969-s1.pdf]

File name: Supplementary Information
Description: Supplementary Notes, Supplementary Figures and Supplementary References

File name: Supplementary Movie 1
Description: Numerical simulation of two populations of motile particles propelling at constant speed and avoiding each other thanks to repulsive torques. Each population targets an opposite direction. After a short transient time the two populations phase separate. Equations of motion described in the main text and Supplementary Equations (1) and (2). Simulation parameters: B = 1, overall packing fraction: πρa2 = 0.65.

File name: Supplementary Movie 2
Description: Numerical simulation of two populations of motile particles propelling at constant speed and avoiding each other thanks to repulsive torques. Each population targets an opposite direction. The two populations mingle and form a homogeneous liquid phase. Equations of motion described in the main text and Supplementary Equations (1) and (2). Simulation parameters: B = 5, overall packing fraction: πρa2 = 0.65.

File name: Peer Review File
Description:

SUPPLEMENTARY NOTE 1: ORIENTATIONAL DIFFUSIVITY

We explain the method used to measure the orientational diffusivity shown in Fig. 1f in the main document. This measurement is reported only for particles in the homogeneous mingled state. The results below correspond to $B = 5$ and $\pi\overline{\rho a^2} \geq 0.3$. We first compute the autocorrelation function of the particle orientation: $\langle \hat{\mathbf{p}}_i(t+T) \cdot \hat{\mathbf{p}}_i(t)\rangle_{i,t}$ where the average is performed both over the particles and the reference time t. This function decays exponentially with the lag time T as shown in Supplementary Figure 1a. $\mathcal{D}_\theta$ is therefore unambiguously defined from the associated decorrelation time from an exponential fit. Repeating the same measurement for different densities we find that $\mathcal{D}_\theta$ increases linearly with $\overline{\rho}$, as shown in Supplementary Figure 1b.

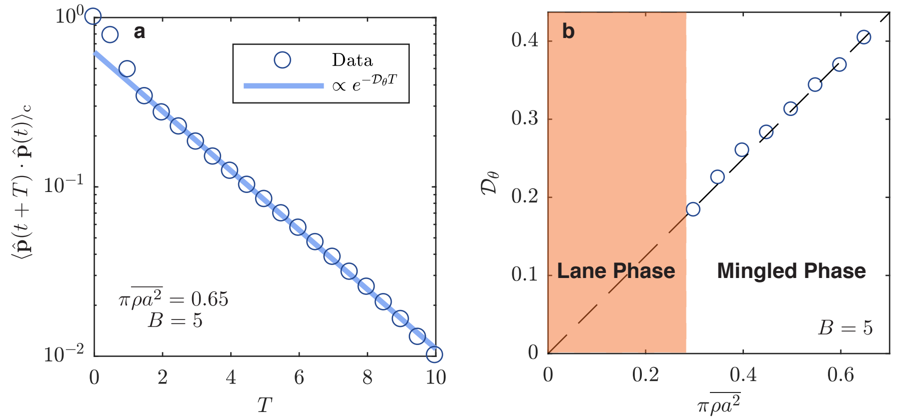

Supplementary Figure 1. Computation of the orientational diffusivity. (a) Log-lin plot of the orientational diffusivity. Dark blue circles: Numerical time autocorrelation function of the unit orientation vector $\hat{\mathbf{p}}$ averaged over all the particles, in the mingled phase at repulsion magnitude $B = 5$ and density $\pi\overline{\rho a^2} = 0.65$. Light blue line: best exponential fit. (b) Orientational diffusivity $\mathcal{D}_\theta$ as a function of the particle area fracion $\pi\overline{\rho a^2}$, at constant repulsion magnitude $B = 5$. It is very nicely approached by a linear fit starting from the origin. The orange area represents the location of the lane phase.

SUPPLEMENTARY NOTE 2: PERSISTENT TRANSVERSE SCATTERING

In this section we study the scattering dynamics of two self-propelled particles of unit radius. For this purpose, let us consider a left mover l and a right mover r, and place the origin of time when contact starts, i.e. when the two interaction disks are tangent. The equations of motion, Supplementary Equations (1) and (2), which we recall below, tell us that the scattering dynamics are fully determined by the magnitude of the repulsive interactions B, the initial orientation of each particle, $\theta_{\rm r}(t=0)$ and $\theta_{\rm l}(t=0)$, and by the initial orientation of the center-to-center vector $\mathbf{r}_{\rm rl}(t=0)$.

$$\dot{\mathbf{r}}_i = \hat{\mathbf{p}}_i, \tag{1}$$

$$\dot{\theta}_i = -\partial_{\theta_i}\mathcal{V}(\theta_i) + \sum_j T_{ij}. \tag{2}$$

Since we assume the particles to be tangent, we are free to place the origin of the $y-$coordinate $y = 0$ halfway between the two particles, making the orientation of the center-to-center vector simply equivalent to the initial vertical position of the right mover: $y_{\rm in} = y_{\rm r}(0) = [y_{\rm r}(0) - y_{\rm l}(0)]/2$, see Supplementary Figure 2a. We are therefore left with a four dimensional parameter space.

A. Frontal collision

As a first approach it is instructive to restrain ourselves to frontal collisions, corresponding to $\theta_{\rm r}(0) = 0$ and $\theta_{\rm l}(0) = \pi$. This situation is particularly relevant to very dilute systems, where the particles have time to perfectly realign with their respective external fields between each collision. The sole impact parameter in this case is the initial vertical distance. Solving the equations of motion, we notice that when the repulsion magnitude increases, so does

the final vertical position $y_{out} = y_r(t \to \infty)$. Moreover, in contrast with what is expected for hardcore repulsion or oppositely driven colloids, it is almost always different from one particle radius (i.e. $y_{out} = 1$), see Supplementary Figure 2b. This can be explained by the competition between the colliding and alignment along the external field. Since no exclusion process is implemented, a weak repulsion implies that the particles realign faster than they deviate from the external field, which results in a small vertical displacement $y_{out} < 1$. Conversely, strong repulsion implies that the particles persist in their deviation for a longer period of time before they realign with the field, leading to $y_{out} > 1$. For $y_{in} > 1$ however, no collision occurs and we simply recover $y_{in} = y_{out}$. Overall, the transverse scattering of opposite self-propelled particles is strongly enhanced by the magnitude of the repulsive torque B.

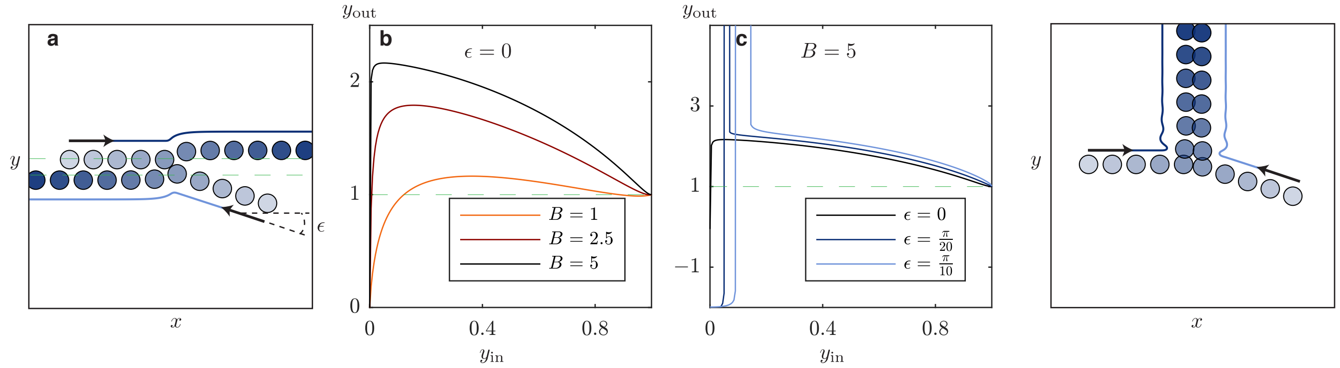

Supplementary Figure 2. Persistent transverse scattering. (a)Typical scattering trajectory for two particles targeting opposite directions ($B = 5$, $\epsilon = \frac{\pi}{10}$ and $y_{in} = 0.75$). The initial and final vertical positions y_{in} and y_{out} are defined in the main text, Fig. 3a. ϵ is the incoming deviation of the left mover. (b) Final vertical position y_{out} as a function of the initial vertical position y_{in} for a left and a right mover experiencing a frontal collision ($\epsilon = 0$) at different values of the repulsion magnitude B. The transverse scattering is always different from the expected value for hard-core collisions (green line), and grows with the repulsion magnitude. (c) Final vertical position y_{out} as a function of the initial vertical position y_{in} at a high repulsion magnitude $B = 5$ for different impact angles ϵ. At a sufficiently high impact angle, the particles can stick to each other and travel upwards or downwards, as pictured in (d). (d) Two particles travelling upwards together ($B = 5$, $\epsilon = \frac{\pi}{10}$ and $y_{in} = 0.125$)

B. Transverse motion

This is, however, just a part of the story. Let us now assume that the collision is not frontal, but that the left mover has an incoming angle of $\theta_l(0) = \pi - \epsilon$. Solving the equations of motion numerically, we find that the two particles can effectively stick to each other and travel upwards or downwards as a bound pair, as illustrated in Supplementary Figures 2c and 2d. This counterintuitive behavior can be however easily explained. Bound pairs form when the two particles turn together either upwards or downwards to avoid each other. In such cases alignement and repulsion compete, and the particles get stuck in this movement until an external event (such as another collision) breaks the pair. The existence of this peculiar solution can be found analytically by looking at the steady state solution of two interacting particles. The $\theta_r = \theta_l = \pm\frac{\pi}{2}$ solution always exists, and it is quite straightforward to show that it is stable for a range of parameters that increases with the magnitude of the interaction B.

SUPPLEMENTARY NOTE 3: LINEARIZED HYDRODYNAMICS IN THE DILUTE LIMIT

The hydrodynamic equation for the velocity field, Eq. (6) in the main text is closed assuming wrapped Gaussian angular fluctuations. This equation admits a constant and homogeneous solution defined by: $\rho_\alpha = \rho_\beta = \rho_0$ and $\mathbf{v}_\alpha = v_0\hat{\mathbf{h}}_\alpha = -\mathbf{v}_\beta$, with the condition $4\mathcal{D}\rho_0 v_0 = (1 - v_0^4)$. In the long wavelength limit, the linear perturbations to this solution $\rho_\alpha = (\rho_0 + \delta\rho_\alpha)$, and $\mathbf{v}_\alpha = (v_0\hat{\mathbf{h}}_\alpha + \delta\mathbf{v}_\alpha)$ obey:

$$\partial_t \delta\rho_\alpha(\mathbf{r}, t) + v_0(\hat{\mathbf{h}}_\alpha \cdot \boldsymbol{\nabla})\delta\rho_\alpha + \rho_0 \boldsymbol{\nabla} \cdot \delta\mathbf{v}_\alpha = 0, \tag{3}$$

and

$$\mathbf{W}_\alpha \cdot \delta \mathbf{v}_\alpha = -\left[\mathbf{A}\cdot\boldsymbol{\nabla}\delta\rho_\alpha + \left(\frac{\rho_0\mathcal{B}}{2}\boldsymbol{\nabla} + \mathcal{D}\rho_0 v_0 \hat{\mathbf{h}}_\alpha\right)\delta\bar{\rho}\right] \tag{4}$$

where we have ignored the fast relaxation of the velocity field and discarded its time derivatives. The hydrodynamic coefficients are defined by: $\mathbf{W}_\alpha = [v_0\mathbf{M}_\alpha + \rho_0(\mathbf{H} + \bar{\rho}_0\mathcal{D}\mathbf{I})]$, $\mathcal{B} = a\mathcal{D}$, and

$$\mathbf{M}_\alpha = \rho_0 v_\alpha^3 \begin{pmatrix} 2\partial_{\rm x} & \partial_{\rm y} \\ -2\partial_{\rm y} & \partial_{\rm x} \end{pmatrix},\ \mathbf{H} = v_0^3 \begin{pmatrix} 2 & 0 \\ 0 & 1 \end{pmatrix} \text{ and } \mathbf{A} = \frac{1}{2}\begin{pmatrix} 1+v_0^4 & 0 \\ 0 & 1-v_0^4 \end{pmatrix}. \tag{5}$$

Eliminating the velocity field from the linearized equations, we find that the large-scale density fluctuations evolve according to:

$$\partial_{\rm t}\delta\rho_\alpha(\mathbf{r},t) + \boldsymbol{\nabla}\cdot(\mathbf{J}_\alpha + \tilde{\mathbf{J}}) = 0, \tag{6}$$

where the two currents are given by:

$$\mathbf{J}_\alpha = \left(v_0\hat{\mathbf{h}}_\alpha - \rho_0(\mathbf{W}_\alpha^{-1}\cdot\mathbf{A})\cdot\boldsymbol{\nabla}\right)\delta\rho_\alpha \tag{7}$$

$$\tilde{\mathbf{J}} = -\rho_0\mathbf{W}_\alpha^{-1}\cdot\left(\mathcal{D}\rho_0 v_0\hat{\mathbf{h}}_\alpha + \frac{\rho_0\mathcal{B}}{2}\boldsymbol{\nabla}\right)\delta\bar{\rho}. \tag{8}$$

The matrix $\mathbf{W}_\alpha$ is readily inverted going to Fourier space. Noting $\mathbf{q}$ the spatial wavevectors we recover the simple expressions provided in the main text:

$$\mathbf{J}_\alpha(\mathbf{q},t) = v_0\hat{\mathbf{h}}_\alpha\delta\rho_\alpha - (\mathbf{D}\cdot\mathbf{q})\delta\rho_\alpha, \tag{9}$$

$$\tilde{\mathbf{J}}(\mathbf{q},t) = -\tilde{v}\hat{\mathbf{h}}_\alpha\delta\bar{\rho} - (\tilde{\mathbf{D}}\cdot\mathbf{q})\delta\bar{\rho}, \tag{10}$$

where the anisotropic diffusivity are diagonal

$$\mathbf{D} = \frac{1}{2}\begin{pmatrix} \frac{1+v_0^4}{\Delta_{\rm x}} & 0 \\ 0 & \frac{1-v_0^4}{\Delta_{\rm y}} \end{pmatrix},\ \tilde{\mathbf{D}} = \frac{1}{2}\begin{pmatrix} \frac{\rho_0\mathcal{B}}{2\Delta_{\rm x}} - \frac{2\mathcal{D}\rho_0 v_0^4}{\Delta_{\rm x}^2} & 0 \\ 0 & \frac{\rho_0\mathcal{B}}{2\Delta_{\rm y}} + \frac{2\mathcal{D}\rho_0 v_0^4}{\Delta_{\rm x}\Delta_{\rm y}} \end{pmatrix} \tag{11}$$

with $\Delta_{\rm x} = 2v_0^3 + 2\mathcal{D}\rho_0$ and $\Delta_{\rm y} = v_0^3 + 2\mathcal{D}\rho_0$ and $\tilde{v} = \frac{\mathcal{D}\rho_0}{\Delta_{\rm x}}v_0$.

SUPPLEMENTARY NOTE 4: LONG-RANGE DENSITY CORRELATIONS

The structural correlations are found adding a conserved δ-correlated noise $\boldsymbol{\xi}_\alpha$ to Supplementary Equation (6):

$$(i\omega\mathbb{I} + \mathbf{R}).\begin{pmatrix} \delta\rho_\alpha \\ \delta\rho_\beta \end{pmatrix} = -\begin{pmatrix} i\mathbf{q}\cdot\boldsymbol{\xi}_\alpha \\ i\mathbf{q}\cdot\boldsymbol{\xi}_\beta \end{pmatrix}, \tag{12}$$

where the linear response matrix $\mathbf{R}$ takes the rather compact form

$$\mathbf{R} = \begin{pmatrix} iq_{\rm x}(v_\alpha - \tilde{v}_\alpha) + (D_{\rm x} + \tilde{D}_{\rm x})q_{\rm x}^2 + (D_{\rm y} + \tilde{D}_{\rm y})q_{\rm y}^2 & -iq_{\rm x}\tilde{v}_\alpha + \tilde{D}_{\rm x}q_{\rm x}^2 + \tilde{D}_{\rm y}q_{\rm y}^2 \\ iq_{\rm x}\tilde{v}_\alpha + \tilde{D}_{\rm x}q_{\rm x}^2 + \tilde{D}_{\rm y}q_{\rm y}^2 & -iq_{\rm x}(v_\alpha - \tilde{v}_\alpha) + (D_{\rm x} + \tilde{D}_{\rm x})q_{\rm x}^2 + (D_{\rm y} + \tilde{D}_{\rm y})q_{\rm y}^2 \end{pmatrix} \tag{13}$$

when defining the hydrodynamics coefficients

$$\tilde{D}_{\rm x} = \frac{1}{2}\left(\frac{\rho_0\mathcal{B}}{2\Delta_{\rm x}} - \frac{2\mathcal{D}\rho_0 v_0^4}{\Delta_{\rm x}^2}\right);\ \tilde{D}_{\rm y} = \frac{1}{2}\left(\frac{\rho_0\mathcal{B}}{2\Delta_{\rm y}} + \frac{2\mathcal{D}\rho_0 v_0^4}{\Delta_{\rm x}\Delta_{\rm y}}\right)$$

$$D_{\rm x} = \frac{1}{2}\left(\frac{1+v_0^4}{\Delta_{\rm x}}\right);\ D_{\rm y} = \frac{1}{2}\left(\frac{1-v_0^4}{\Delta_{\rm y}}\right).$$

The density fluctuations are readily computed within this linear response framework.

$$\langle|\delta\rho_\alpha(\mathbf{q})|^2\rangle = C_0(\mathbf{q})\left(\frac{\left[q_{\rm x}^2(\tilde{D}_{\rm x} + D_{\rm x}) + q_{\rm y}^2(\tilde{D}_{\rm y} + D_{\rm y})\right]^2 + [q_{\rm x}(v_0 - \tilde{v})]^2}{\left[q_{\rm x}^2 D_{\rm x} + q_{\rm y}^2 D_{\rm y}\right]\left[q_{\rm x}^2(2\tilde{D}_{\rm x} + D_{\rm x}) + q_{\rm y}^2(2\tilde{D}_{\rm y} + D_{\rm y})\right] + q_{\rm x}^2 v_0(v_0 - 2\tilde{v})}\right) \tag{14}$$

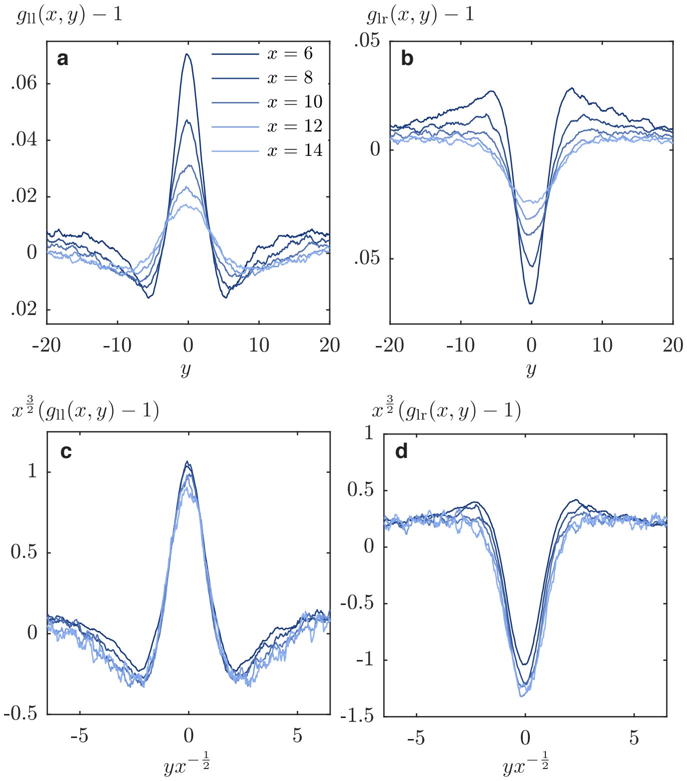

Supplementary Figure 3. Density correlation functions evaluated at a fixed values of x plotted versus the distance in the transverse direction y. Simulations performed deep in the homogeneous phase ($B = 5$ and $\pi\overline{\rho a^2} = 0.65$). (a) Bare pair correlation functions functions for particles of the same population $g_{ll}(x, y)$ (left movers). (b) Bare pair correlation function for particles belonging to different populations $g_{lr}(x, y)$. (c) and (d) Collapse of the pair correlations once rescaled by the universal $x^{-3/2}$ power law and plotted as a function of the rescaled distance $y/\sqrt{x}$. The good collapse of the rescaled curves supports the validity of the scaling deduced from the linearized fluctuating hydrodynamics.

and

$$\langle\delta\rho_\alpha(\mathbf{q})\delta\rho_\beta(-\mathbf{q})\rangle = C_0(\mathbf{q})\left(\frac{\left[q_x^2\tilde{D}_x + q_y^2\tilde{D}_y - iq_x\tilde{v}_\alpha\right]\left[q_x^2(\tilde{D}_x + D_x) + q_y^2(\tilde{D}_y + D_y) - iq_x(v_\alpha - \tilde{v}_\alpha)\right]}{\left[q_x^2 D_x + q_y^2 D_y\right]\left[q_x^2(2\tilde{D}_x + D_x) + q_y^2(2\tilde{D}_y + D_y)\right] + q_x^2 v_0(v_0 - 2\tilde{v})}\right) \quad (15)$$

with

$$C_0(\mathbf{q}) = \frac{q^2 T}{q_x^2(\tilde{D}_x + D_x) + q_y^2(\tilde{D}_y + D_y)} \quad (16)$$

where, for simplicity and with no loss of generality, the added noise is chosen to be isotropic. An anisotropy would simply write $q_x^2 T_x + q_y^2 T_y$ and not change any of the results. One can readily recognize in the prefactor $C_0(\mathbf{q})$ the density autocorrelation function of an out-of-equilibrium anisotropic system with conserved dynamics and noise, as described in [1].

As we are interested in the long distance behavior of the density autocorrelation functions i.e. the limit $\mathbf{q} \to \mathbf{0}$, we focus on the simpler form they take at lowest order in q_x and q_y:

$$\langle|\delta\rho_\alpha(\mathbf{q})|^2\rangle = C_0(\mathbf{q})\left(\frac{q_y^4(\tilde{D}_y + D_y)^2 + q_x^2(v_0 - \tilde{v})^2}{q_y^4 D_y(2\tilde{D}_y + D_y) + q_x^2 v_0(v_0 - 2\tilde{v})}\right) \quad (17)$$

$$\langle\delta\rho_\alpha(\mathbf{q})\delta\rho_\beta(-\mathbf{q})\rangle = C_0(\mathbf{q})\left(\frac{\left[q_y^2\tilde{D}_y - iq_x\tilde{v}_\alpha\right]\left[q_y^2(\tilde{D}_y + D_y) - iq_x(v_\alpha - \tilde{v}_\alpha)\right]}{q_y^4 D_y(2\tilde{D}_y + D_y) + q_x^2 v_0(v_0 - 2\tilde{v})}\right). \quad (18)$$

The long wavelength limit of these nonanalytic correlations is not uniquely defined as already pointed out in [2]. We therefore have to specify the way we take this limit. If we keep both wave vectors $q_{\rm x}$ and $q_{\rm y}$ of the same order as they go to zero, we obtain $\langle|\delta\rho_\alpha(\mathbf{q})|^2\rangle \propto C_0(\mathbf{q})$, yielding an asymptotic behavior in real space $|g_{\alpha\beta}(x,0)-1| \sim x^{-2}$ [1], not consistent with our numerical findings. This is not surprising since in this given limit, all the specifities of our system, namely the interactions and the alignment field, are thrown out of the equations. However, the specific long-range limit $q_{\rm x} \sim q_{\rm y}^2$ simplifies the prefactor into $C_0(\mathbf{q}) \sim T/(\tilde{D}_{\rm y}+D_{\rm y})$ and preserves the main ingredients of our system. $\langle|\delta\rho_\alpha(\mathbf{q})|^2\rangle$ then has the same analytic expression as that derived for oppositely driven colloids in [2] and the cross-correlation $\langle\delta\rho_\alpha(\mathbf{q})\delta\rho_\beta(-\mathbf{q})\rangle$ becomes a linear combination of the auto- and cross-correlation functions derived in [2]. They therefore both have the same asymptotic behavior in real space: $|g_{\alpha\beta}(x,0)-1| \sim x^{-3/2}$ which correctly accounts for our numerical findings as evidenced in Fig. 4b in the main text. In addition, Fourier transforming Supplementary Equations (17) and (18), we find that they are both homogeneous functions obeying $|1-g_{\alpha\beta}(x\to\infty,y)| \sim x^{-\frac{3}{2}}\mathcal{C}(yx^{-1/2})$, again in agreement with our numerical simulations as shown in Supplementary Figure 3. This emphasizes the universality of the results obtained by linear perturbation of the kinetic theory.

SUPPLEMENTARY NOTE 5: HYDRODYNAMIC THEORY IN THE LARGE DENSITY LIMIT

The hydrodynamic theory exploited in the previous sections was derived using a Boltzmann ansatz valid in the dilute limit. However the homogeneous mingled state also extends in the high density range. In order to further establish the robustness of the long-range structural correlation in this dynamical state, we here establish the hydrodynamic description of the interacting populations in the limit of very large densities. In order to do so we use a mean-field approach. We first write the continuity equation for the $2N$-point distribution function $\psi^{(2N)}(\mathbf{r}_{\alpha 1},...,\mathbf{r}_{\alpha N},\theta_{\alpha 1},...\theta_{\alpha N},\mathbf{r}_{\beta 1},...,\mathbf{r}_{\beta N},\theta_{\beta 1},...\theta_{\beta N})$

$$\partial_{\rm t}\psi^{(2N)} + \sum_{i=\alpha,\beta}\boldsymbol{\nabla}_i.(\hat{\mathbf{p}}_i\psi^{(2N)}) + \sum_{i=\alpha,\beta}\partial_{\theta_i}\left(\dot{\theta}_i\psi^{(2N)}\right) = 0. \tag{19}$$

Integrating over all the particles but one, and using the equation of motion Supplementary Equation (2), we obtain a relation respectively between the one-point function, $\psi_\alpha(\mathbf{r}_\alpha,\theta_\alpha,t)$, and the two-point functions $\psi^{(2)}_{\alpha\alpha'}$ and $\psi^{(2)}_{\alpha\beta}$.

$$\partial_{\rm t}\psi_\alpha + \boldsymbol{\nabla}\cdot(\hat{\mathbf{p}}\psi_\alpha) + \partial_\theta\left[\partial_\theta\left(\hat{\mathbf{p}}\cdot\hat{\mathbf{h}}_\alpha\right)\psi_\alpha\right] = -\partial_\theta\left(\int d\mathbf{r}'_\alpha d\theta'_\alpha T_{\alpha\alpha'}\psi^{(2)}_{\alpha\alpha'} + \int d\mathbf{r}_\beta d\theta_\beta T_{\alpha\beta}\psi^{(2)}_{\alpha\beta}\right), \tag{20}$$

within a mean field hypothesis, a priori valid in the limit of infinite densities, we assume the factorization of the two-point functions:

$$\psi^{(2)}_{\alpha\beta} = \psi^{(1)}_\alpha\psi^{(1)}_\beta, \tag{21}$$

which yields

$$\partial_{\rm t}\psi_\alpha + \boldsymbol{\nabla}\cdot(\hat{\mathbf{p}}\psi_\alpha) + \partial_\theta\left[\partial_\theta\left(\hat{\mathbf{p}}\cdot\hat{\mathbf{h}}_\alpha\right)\psi_\alpha\right] = -\partial_\theta\left(\psi_\alpha\left[\int d\mathbf{r}'_\alpha d\theta'_\alpha T_{\alpha\alpha'}\psi_{\alpha'} + \int d\mathbf{r}_\beta d\theta_\beta T_{\alpha\beta}\psi_\beta\right]\right). \tag{22}$$

Integrating this equation once with respect to θ yield the mass conservation equation. Multiplying Supplementary Equation (22) by $\hat{\mathbf{p}}$ before integrating over θ, we obtain a differential equation for the (non conserved) velocity field

$$\partial_t(\rho_\alpha\mathbf{v}_\alpha) + \boldsymbol{\nabla}\cdot\left[\rho_\alpha\left(\frac{\mathbb{I}}{2}+\mathbf{Q}_\alpha\right)\right] = \rho_\alpha\left(\frac{\mathbb{I}}{2}-\mathbf{Q}_\alpha\right)\cdot\left(\hat{\mathbf{h}}_\alpha - \mathcal{B}\boldsymbol{\nabla}\overline{\rho}\right) \tag{23}$$

where $\mathcal{B}$ is a hydrodynamic coefficient which increases with the magnitude of the repulsion between the motile particles. We shall note that Supplementary Equation (23) does not include any collision-induced diffusion term, unlike what was found in the dilute limit. This result is not surprising as collision-induced diffusivity arises from local density fluctuations that are discarded by our mean-field approximations. However, we can add a phenomenological angular diffusion to Supplementary Equation (23) on the basis of our numerical observations of the form $(\mathcal{D}\overline{\rho})\rho_\alpha\mathbf{v}_\alpha$ (see Supplementary Figure 1b). The resulting hydrodynamic equations have the very same form as Supplementary Equations (6) to (11). Not surprisingly, conducting the same fluctuating hydrodynamic analysis we find the same density correlations in the (stable) homogeneous state. Supplementary Equations (17) and (18) are merely modified by the expressions of the effective anisotropic diffusivities, not affecting the long range correlations in any way:

$$\tilde{D}_{\rm x} \to \frac{1}{2}\left(\rho_0\mathcal{B}\frac{1-v_0^4}{\Delta_{\rm x}} - \frac{2\mathcal{D}\rho_0 v_0^4}{\Delta_{\rm x}^2}\right) \text{ and } \tilde{D}_{\rm y} \to \frac{1}{2}\left(\rho_0\mathcal{B}\frac{1+v_0^4}{\Delta_{\rm y}} + \frac{2\mathcal{D}\rho_0 v_0^4}{\Delta_{\rm x}\Delta_{\rm y}}\right). \tag{24}$$

SUPPLEMENTARY REFERENCES

[1] Grinstein G., Lee D. H., and Sachdev S., "Conservation laws, anisotropy, and "self-organized criticality" in noisy nonequilibrium systems," Phys. Rev. Lett. **64**, 1927–1930 (1990).
[2] Poncet A., Bénichou O., Démery V., and Oshanin G., "Universal long ranged correlations in driven binary mixtures," Phys. Rev. Lett. **118**, 118002 (2017).
